# *In situ* activation and monitoring of the evolution of the intracellular caspase family†
†Electronic supplementary information (ESI) available: Experimental details and supplementary figures. See DOI: 10.1039/c5sc00471c



**DOI:** 10.1039/c5sc00471c

**Published:** 2015-03-26

**Authors:** Lei Zhang, Jianping Lei, Jintong Liu, Fengjiao Ma, Huangxian Ju

**Affiliations:** a State Key Laboratory of Analytical Chemistry for Life Science , School of Chemistry and Chemical Engineering , Nanjing University , Nanjing 210093 , P. R. China . Email: jpl@nju.edu.cn ; Email: hxju@nju.edu.cn ; Fax: +86 25 83593593 ; Tel: +86 25 83593593

## Abstract

The evolution of the intracellular caspase family is crucial in cell apoptosis. To evaluate this process, a universal platform of *in situ* activation and monitoring of the evolution of intracellular caspase is designed. Using well-known gold nanostructure as a model of both nanocarrier and matter inducing the cell apoptosis for photothermal therapy, a nanoprobe is prepared by assembly of two kinds of dye-labelled peptides specific to upstream caspase-9 and downstream caspase-3 as the signal switch, and folic acid as a targeting moiety. The energy transfer from dyes to the gold nanocarrier at two surface plasmon resonance absorption wavelengths leads to their fluorescence quenching. Upon endocytosis of the nanoprobe to perform the therapy against cancer cells, the peptides are successively cleaved by intracellular caspase activation with the evolution from upstream to downstream, which lights up the fluorescence of the dyes sequentially, and can be used to quantify both caspase-9 and caspase-3 activities in cancer cells and to monitor their evolution in living mice. The recovered fluorescence could also be used to assess therapeutic efficiency. This work provides a novel powerful tool for studying the evolution of the intracellular caspase family and elucidating the biological roles of caspases in cancer cell apoptosis.

## Abstract 2

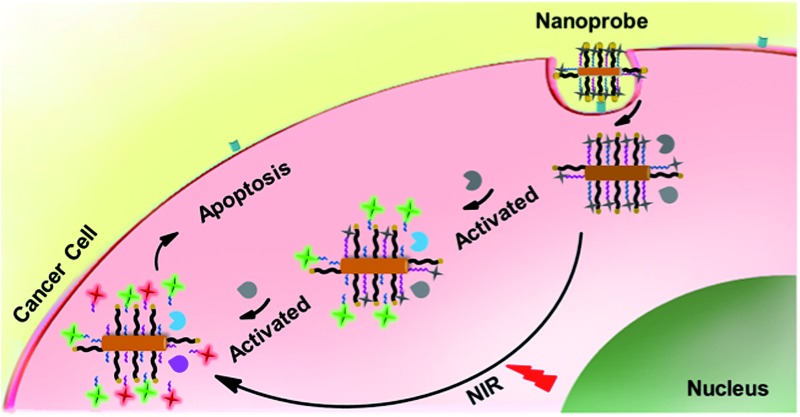
An intergrated nano-platform is designed to achieve *in situ* activation, monitoring and signal feedback of the caspase family evolution from upstream to downstream.

## Introduction

Caspases are a family of cysteine–aspartic proteases that are only activated during cell apoptosis, and can be used as feedback markers of cell death.^1^ Caspase-controlled apoptosis has a characteristic enzyme cascade, which involves multiple caspases at different stages and pathways.^2^ Upstream caspase, such as caspase-9 (casp-9), plays a central role in the induction of apoptosis, while downstream caspase such as caspase-3 (casp-3) is critical for carrying out the final step of cell apoptosis. Thus the evaluation of the intracellular caspase family is essential to elucidate the cell apoptosis process. Indeed, many fluorescent probes have been developed for imaging of caspase activity in living cells and animals,^3^ and real-time monitoring of caspase cascade activation by diverse pairs of dyes and corresponding quenchers.^4^ However, besides the sensing probes, some additional inducers are usually needed to activate intracellular caspase activity.^4,5^ Thus an apoptosis sensor has developed for *in situ* activation and imaging of intracellular casp-3 using aggregation-induced emission.^6^ A platform for *in situ* activation and monitoring the evolution of caspase family during cell apoptosis is still an urgent need.

Noble metal nanostructures with good biocompatibility have received considerable research interest due to their strong absorption in the near-infrared (NIR) region and high photothermal conversion efficiency.^7^ Herein, using gold nanorods (AuNR) as the model of both nanocarrier and matter inducing the cell apoptosis, which was found to be able to quench simultaneously two kinds of dyes at two unique surface plasmon resonance (SPR) absorption wavelengths, a versatile nanoprobe was designed for *in situ* activation and monitoring of the evolution of the caspase family from upstream to downstream *via* NIR photothermal treatment.

**Scheme 1:**
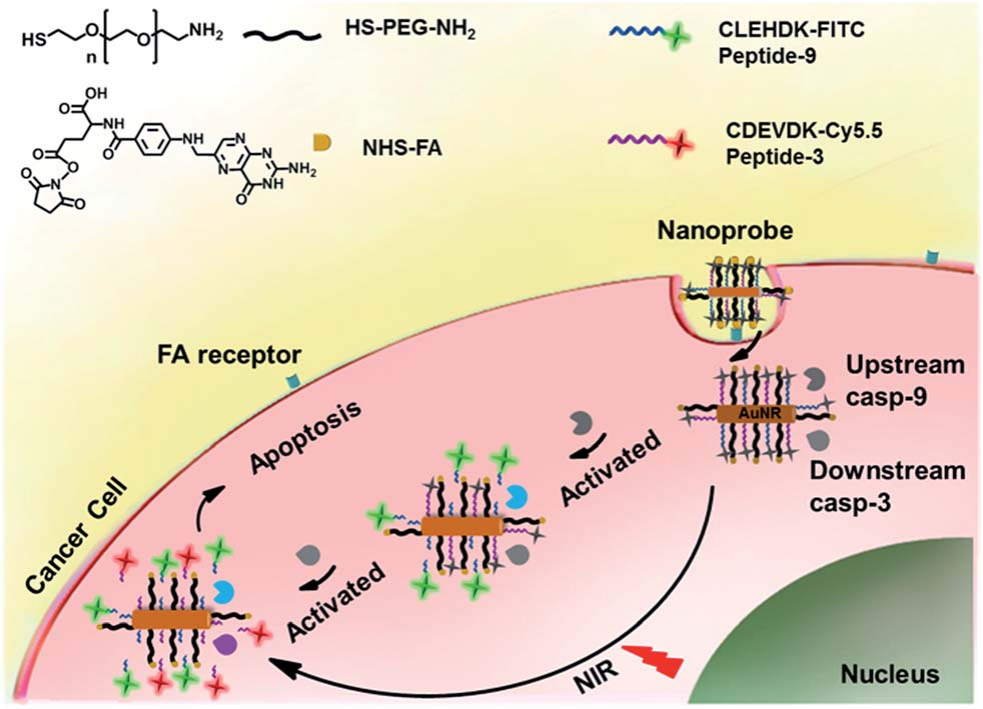
Schematic illustration of an integrated platform for *in situ* monitoring of the evolution of the caspase family activated *via* real-time NIR photothermal therapy.

The nanoprobe was prepared by assembling a fluorescein isothiocyanate (FITC)-labelled peptide specific to casp-9 (peptide-9) and cyanine-5.5 (Cy5.5)-labelled peptide specific to casp-3 (peptide-3) as signal switches and recognition elements, and folic acid (FA) as a target specific moiety on AuNR (Scheme 1). Their fluorescence was initially quenched *via* energy transfer from FITC and Cy5.5 to AuNR with transverse and longitudinal SPR absorption, respectively. Upon endocytosis of the nanoprobe and NIR irradiation, cell apoptosis was encouraged by the photothermal effect and thus the peptide could be cleaved successfully by the corresponding activated caspase from upstream casp-9 to downstream casp-3, which released the dyes from the nanocarrier for fluorescent imaging. The turn-on signals provided an efficient way for quantification of both caspa-9 and casp-3 activities in cancer cells and monitoring of their evolution in living mice. Since caspase activity is the marker of cell apoptosis, the fluorescence response could also be used to monitor therapeutic efficacy in real time.

## Results and discussion

### Characterizations of the nanoprobe

Gold nanocarriers were synthesized according to a typical method of seed-mediated growth and well characterized (see ESI, Fig. S1A and S2†).^8^ For efficient preparation of the nanoprobe, HS-poly(ethylene glycol)-NH_2_ was used to protect the nanorod from aggregation and bind efficiently *N*-hydroxysuccinimide (NHS)-functionalized FA *via* a typical amide reaction (see ESI, Fig. S3†).^9^ After functionalization with dye-labelled peptides and PEG, a slight red shift of the characteristic absorption at 787 nm was observed in the UV-vis spectra (Fig. S2A†), while the binding of NHS-FA to the PEG produced a characteristic absorption peak of FA at 280 nm.^10^ The functionalization did not change their surface profile (Fig. S1B†). In addition, compared with raw nanorods, the nanoprobe showed an Au–Br Raman peak with the shift from 180 cm^–1^ to 261 cm^–1^ (Fig. S2B†), and the surface changed to a negative *ζ* potential (Fig. S2C†). These results indicated that the nanoprobe was synthesized successfully with good-dispersibility and excellent optical properties for fluorescent detection and imaging.

### *In vitro* detection of casp-9 and casp-3 activities

**Fig. 1:**
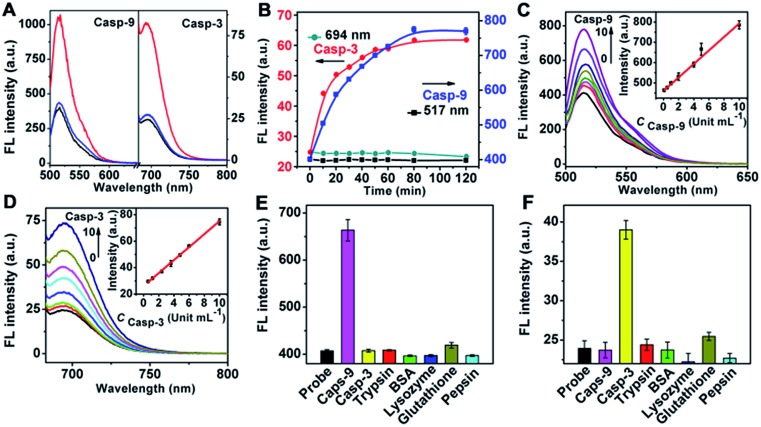
(A) Fluorescence spectra of nanoprobe (OD_787nm_ = 0.5, black) and its mixtures with casp-9 or casp-3 (20 Unit mL^–1^) in the absence (red) and presence (blue) of casp-9 or casp-3 inhibitor (10 μM). (B) Time-dependent fluorescence responses of nanoprobe (10 μL) in the absence (black) and presence (blue) of casp-9 (8.0 Unit mL^–1^) at 517 nm, and in the absence (green) and presence (red) of casp-3 (8.0 Unit mL^–1^) at 694 nm. (C and D) Fluorescence spectra of nanoprobe (10 μL) after incubation with (C) 0, 0.1, 0.5, 1.0, 2.0, 4.0, 5.0 and 10 Unit mL^–1^ casp-9 and (D) 0, 0.6, 1.2, 2.4, 3.6, 4.8, 6.0 and 10 Unit mL^–1^ casp-3 for 80 min. Insets: plots of FL intensity *vs.* casp-9 and casp-3 concentration. Fluorescent response of nanoprobe at (E) 517 and (F) 694 nm to casp-9 (5.0 Unit mL^–1^), casp-3 (5.0 Unit mL^–1^), glutathione (1.0 mM) and other proteins (1.0 μM).

To test the validity of the nanoprobe to caspase, *in vitro* enzymatic assays were performed with recombinant caspase proteins. Since the emission of FITC and Cy5.5 overlapped with the transverse and longitudinal SPR absorption of the gold nanorod (see ESI, Fig. S4†), their fluorescence (FL) was quenched *via* FRET (see ESI, Fig. S5†), respectively. After incubating the mixture of the nanoprobe and recombinant caspase proteins in caspase assay buffer at 37 °C, the FL was significantly enhanced at 517 and 694 nm, respectively, indicating that the enzymatic reaction released the dyes from the gold nanostructure, which could be inhibited by the specific inhibitors of casp-9 or casp-3 (Fig. 1A). At the optimized reaction time of 80 min (Fig. 1B), the FL intensity increased linearly with the enhancing concentration of caspase (Fig. 1C and D). The cleavage reaction showed good specificity to caspase against other interferents (Fig. 1E and F), leading to a method for the detection of caspase activity.

**Fig. 2:**
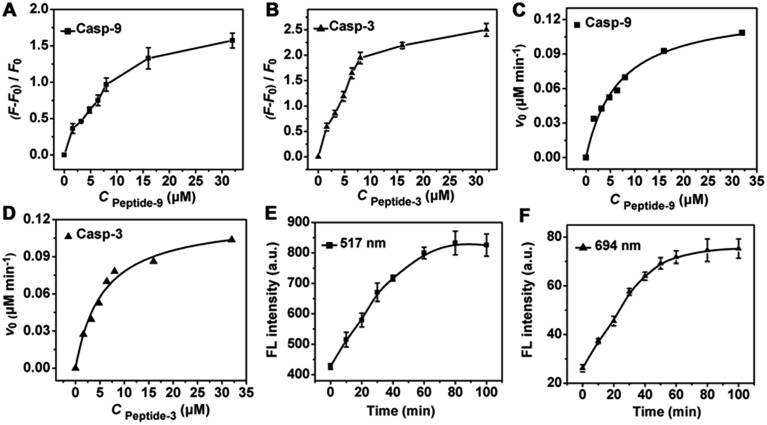
Plots of (*F* – *F*_0_)/*F*_0_ *vs.* concentration of (A) peptide-9 and (B) peptide-3 loaded on nanoprobes, where *F*_0_ and *F* are the fluorescence intensity of nanoprobe and the mixture of nanoprobe with target caspase (10 Unit mL^–1^) after incubation for 80 min at 37 °C, respectively. Caspase enzymatic kinetics assay of (C) casp-9 (10 Unit mL^–1^) and (D) casp-3 (10 Unit mL^–1^) with increasing substrate concentration. Time-dependence of the fluorescent response of nanoprobe (10 μL) in apoptotic HeLa cell lysate at (E) 517 nm and (F) 694 nm.

The amounts of peptide-9 and peptide-3 loaded on the nanocarrier were determined to be 1.01 × 10^4^ and 9.73 × 10^3^, respectively (see ESI, Fig. S6†). The kinetic analysis of cleavage reactions was carried out by incubating casp-9 or casp-3 with the increasing concentration of nanoprobe at 37 °C. The Michaelis constants, *K*_M_, of casp-9 and casp-3 were calculated to be 6.70 ± 0.4 and 5.58 ± 0.3 μM, and the kinetic constants, *k*_cat_, were 1.69 and 1.62 s^–1^, respectively (Fig. 2A–D). The *k*_cat_ value for casp-3 was comparable to the value reported with another fluorescent probe,^11^ and the *K*_M_ value was lower than the 12.7 μM of a commercial substrate for casp-3,^12^ indicating the better affinity between the nanoprobe and caspase.

Owing to the essential roles of caspases in cell apoptosis, the selectivity of nanoprobes in monitoring the activity of caspase in complex cellular samples was examined. HeLa cell lysates were collected after treatment with a commonly used cell apoptosis inducer (staurosporine, 2 μM) to activate the caspase. Time-dependent fluorescence at both 517 and 694 nm with excitation wavelengths of 490 and 675 nm was detected during the incubation of the lysate with nanoprobe, respectively (Fig. 2E and F), which showed the acceptable selectivity of the nanoprobe for intracellular activated casp-9 and casp-3.

### Monitoring the evolution of the intracellular caspase family

Prior to intracellular usage, the cytotoxicity of nanoprobe and NIR irradiation (808 nm) was examined with an MTT assay (see ESI, Fig. S7†). After incubation with different amounts of nanoprobe for 3 h or treatment with NIR for 50 min, HeLa cells still maintained a high viability, indicating the good biocompatibility of nanoprobe and the low cytotoxicity of NIR irradiation itself. Compared with HaCat normal cells, the nanoprobe-transfected HeLa cells showed obvious apoptosis after NIR irradiation at 4 W cm^–2^ for 10 min. The nanoprobe could enter HeLa cells *via* FA receptor-mediated endocytosis and accumulate in the cytoplasm outside of cell nucleus (see ESI, Fig. S8 and S9†), which was also verified by TEM image (see ESI, Fig. S10†). The weak fluorescence of FITC could be observed in MCF-7 and HeLa cells, while no change was observed in A549 cells, confirming FA receptor-mediated internalization of the nanoprobe.

**Fig. 3:**
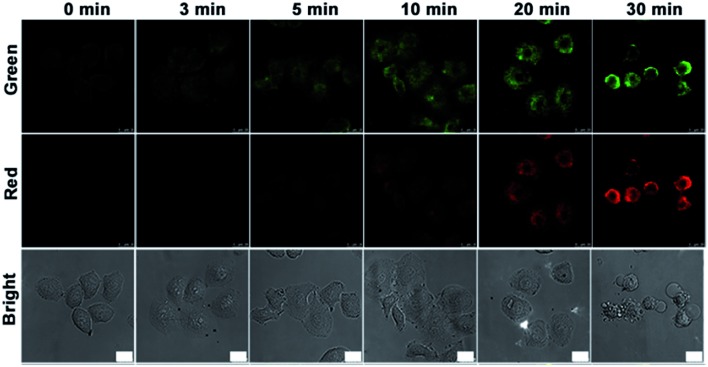
Confocal fluorescence images of HeLa cells incubated with the nanoprobe (10 μL, OD_787nm_ = 0.5) for 3 h and then treated with NIR irradiation at 4 W cm^–2^ for different times. Green fluorescence at *λ*_ex/em_ of 488/500–560 nm. Red fluorescence at *λ*_ex/em_ of 633/680–740 nm. Scale bars, 25 μm.

To employ the probe to monitor the evolution of the caspase family from upstream to downstream during therapy, HeLa cells were seeded in a confocal dish for 24 h. After incubation with the nanoprobe for 3 h and then treatment with NIR irradiation for different times, the HeLa cells were then studied with confocal fluorescence imaging (Fig. 3). Little fluorescence was observed in the nanoprobe transfected HeLa cells before NIR irradiation. After NIR irradiation for 3 min the fluorescence of FITC (green) in HeLa cells first appeared, and then increased gradually with the progression of cell apoptosis induced by the photothermal effect of the gold nanocarrier, while the fluorescence of Cy5.5 (red) was observed after NIR irradiation for 10 min, indicating that casp-9 was activated in the initial stage of cell apoptosis, which thus played the role of initiator in the caspase family. With the deepening of the degree of cell apoptosis, casp-3 was activated to show the fluorescence of Cy5.5. After irradiation for 30 min, apoptotic HeLa cells showed shrinkage with maximum fluorescence intensity in both the green and red channels.

**Fig. 4:**
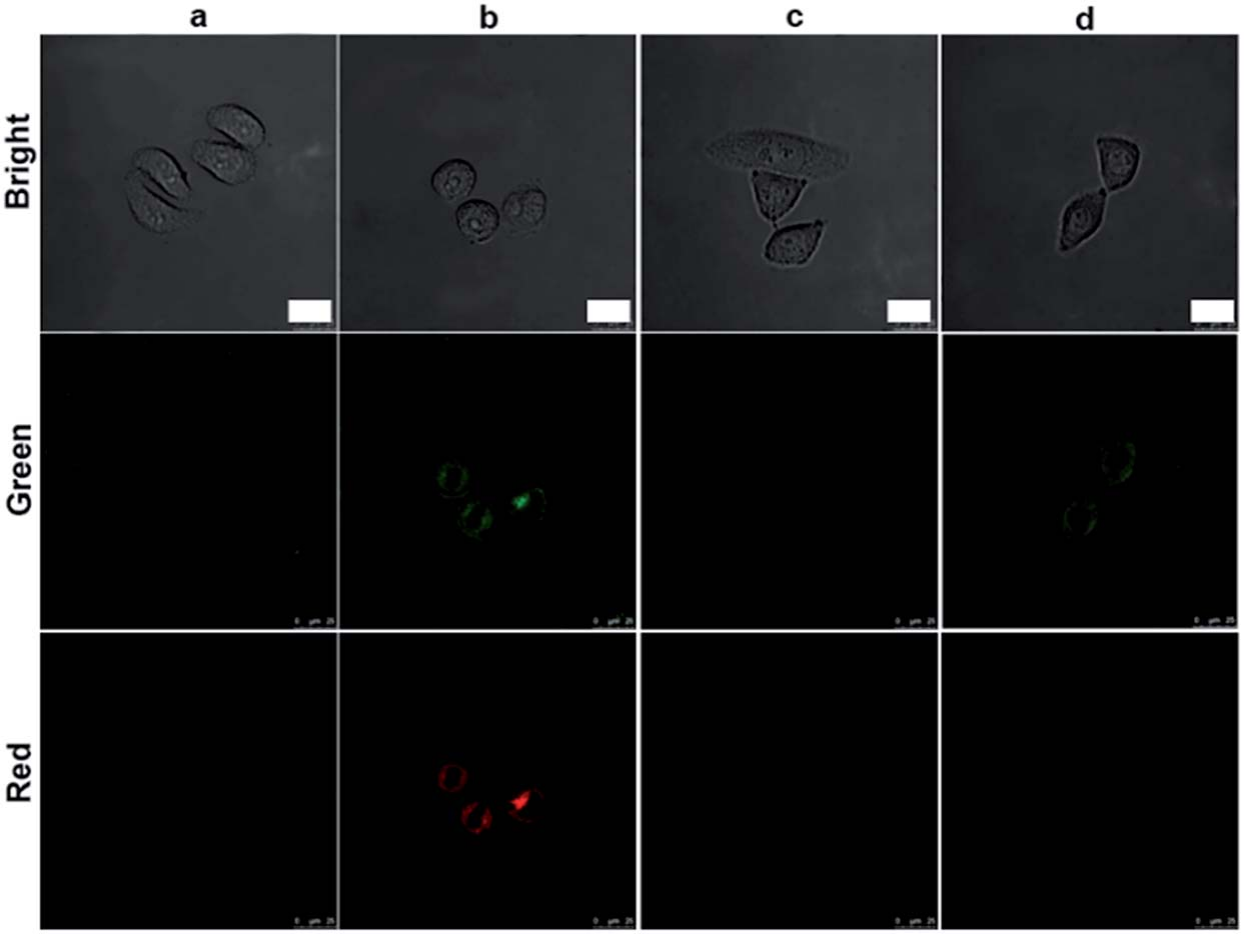
Confocal fluorescence images of HeLa cells incubated with 10 μL nanoprobe (a) before and (b) after NIR irradiation for 20 min, and treated with (c) casp-9 and (d) casp-3 inhibitor for 6 h and then NIR irradiation for 20 min. Green fluorescence at *λ*_ex/em_ of 488/500–560 nm. Red fluorescence at *λ*_ex/em_ of 633/680–740 nm. Scale bars, 25 μm.

**Fig. 5:**
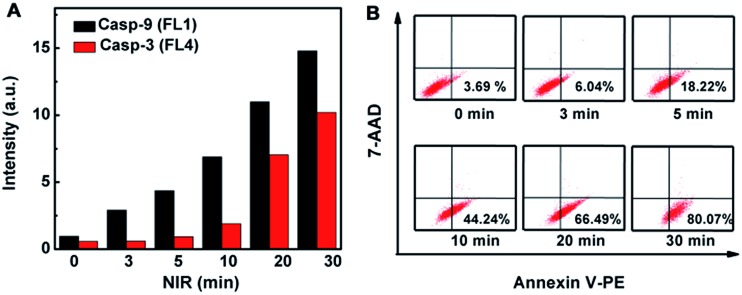
(A) Flow cytometric detection of HeLa cells after incubation with the nanoprobe (50 μL, OD_787nm_ = 0.5) for 3 h and then NIR irradiation for different times. (B) Flow cytometric analysis of the same treated HeLa cells using apoptosis kit with the dual fluorescence of Annexin V-PE/7-AAD.

The initiator role of casp-9 in the evolution of the caspase family could be confirmed by inhibitor treatment (Fig. 4). After the probe-transfected HeLa cells were treated with casp-3 inhibitor, they showed the green fluorescence of FITC, and the red Cy5.5 disappeared, indicating the effective inhibition of the inhibitor to casp-3, which did not affect the activation of casp-9 to release FITC from the nanoprobe. Contrarily, after the probe-transfected HeLa cells were treated with casp-9 inhibitor, both the green fluorescence and the following red fluorescence disappeared. Thus the activation of casp-3 depended on casp-9, which demonstrated the involvement of the mitochondrial apoptotic pathway.^13^ The evolution was also validated by flow cytometric assays, which showed the increasing fluorescence of FITC and Cy5.5 (Fig. 5A) as the apoptosis percentage rose (Fig. 5B).

The mitochondrial pathway of apoptosis was confirmed using Rhodamine 123 staining (see ESI, Fig. S11†), which is readily sequestered by living mitochondria in cells undergoing apoptosis.^14^ Furthermore, both the apoptotic detection kit with flow cytometry (Fig. 5B) and fluorescence imaging of HeLa cells stained with 4′,6-diamidino-2-phenylindole (DAPI) dye, specific for the cell nucleus (see ESI, Fig. S12†), verified the caspase-dependent early apoptosis through mitochondrial pathway.^15^

**Fig. 6:**
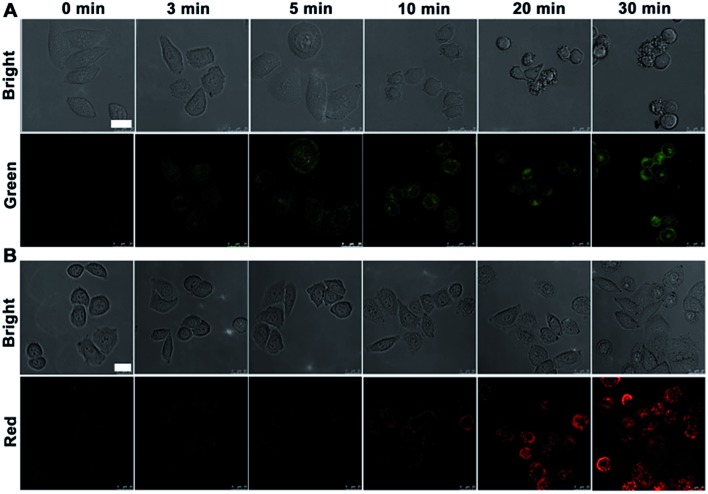
Confocal fluorescence images of HeLa cells after incubation with (A) nanoprobe-9 (10 μL) and (B) nanoprobe-3 (10 μL) for 3 h and then NIR irradiation for different times. Green fluorescence at *λ*_ex/em_ of 488/500–560 nm. Red fluorescence at *λ*_ex/em_ of 633/680–740 nm. Scale bars, 25 μm.

To verify further the application of the designed nanoprobe in monitoring the evolution of the caspase family, nanoprobe-9 and nanoprobe-3 were also synthesized (see ESI†). After incubating HeLa cells with these probes for 3 h at 37 °C, the cells were treated with NIR irradiation for different times. Consistent with the appearance shown in Fig. 3, the nanoprobe-9 transfected HeLa cells showed the green fluorescence at 3 min post-irradiation followed with casp-9 activation (Fig. 6A), while the nanoprobe-3 transfected cells showed red fluorescence from 10 min due to the activation of casp-3 (Fig. 6B). The specificity of the intracellular cleavage reaction was demonstrated by immunofluorescence imaging (see ESI, Fig. S13†). An excellent overlap was observed both between the green fluorescence of nanoprobe-9 and the immunofluorescence signal of casp-9, and the red fluorescence of nanoprobe-3 and immunofluorescence signal of casp-3, suggesting intracellular caspase-specific activation and imaging.

### Quantification of two intracellular caspases

**Fig. 7:**
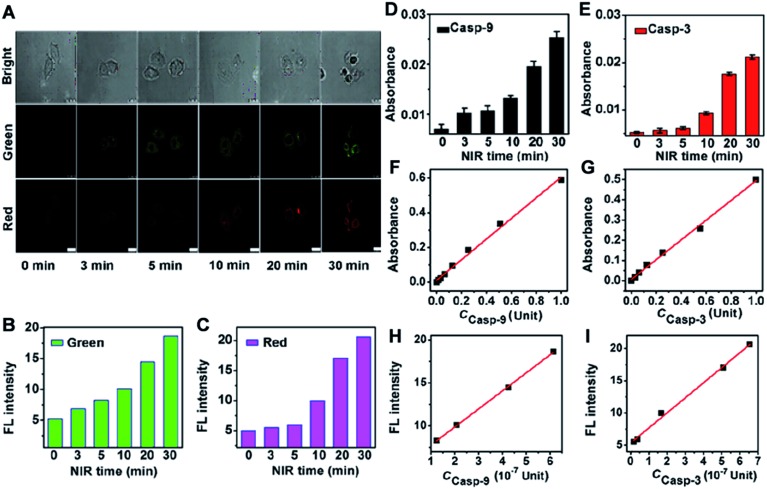
(A) Confocal fluorescence images of HeLa cells treated with 10 μL nanoprobe (OD_787nm_ = 0.5) for 3 h and then NIR irradiation for different times. Green fluorescence at *λ*_ex/em_ of 488/500–560 nm; red fluorescence at *λ*_ex/em_ of 633/680–740 nm. Scale bars, 25 μm. (B and C) FL intensity in single cell area obtained from (A) with Leica software. (D and E) *In vitro* casp-9 and casp-3 kit analysis of cell extracts with the standard curves shown in (F) and (G). (H and I) Calibration curves for activity detection of intracellular casp-9 and casp-3.

The proposed probe could be used to quantify the activities of two intracellular caspases. To obtain the calibration curve, HeLa cells (5.0 × 10^4^) were incubated with the nanoprobe for 3 h and then treated with NIR irradiation with increasing time to obtain the confocal fluorescence images. The FL intensity was measured in the cell area with Leica software (Fig. 7A–C). The corresponding casp-9 and casp-3 activities of the treated HeLa cells were detected *via in vitro* casp-9 and casp-3 kit analysis of the cell extracts using their standard curves (Fig. 7D–G). The obtained caspase activities (*c*) were then used to obtaining the calibration curve for quantification of casp-9 and casp-3 activities in a single cell from the FL intensity (Fig. 7H and I). The plots of FL intensity (FI) *vs. c* (10^–7^ Unit) in single cell followed the linear regression equations of FI = 5.67 + 2.10 × *c* for casp-9 and FI = 5.33 + 2.33 × *c* for casp-3. The average activity of casp-9 and casp-3 in a single HeLa cell was 4.25 and 5.09 × 10^–7^ Unit after the therapy-induced apoptosis. Therefore, the proposed strategy possessed the applicability to monitor the change of intracellular caspase activity.

### Evaluation of therapeutic efficiency in real-time

**Fig. 8:**
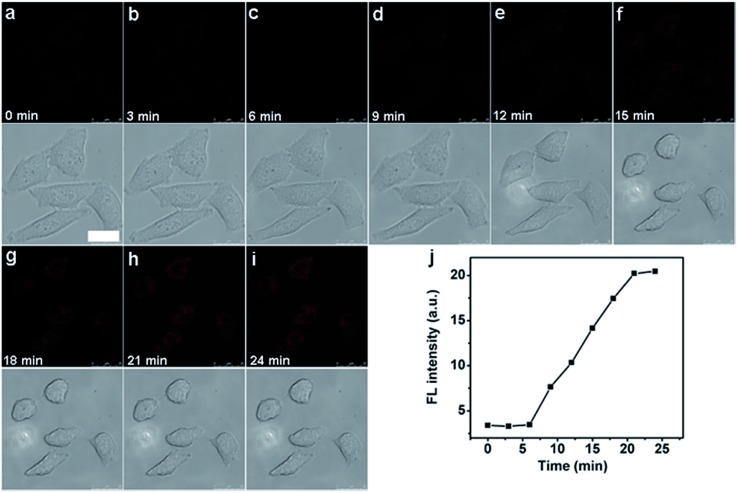
Real-time monitoring of fluorescence and morphology of HeLa cells treated with nanoprobe (10 μL) for 3 h and then NIR irradiation at a power density of 4 W cm^–2^ for different times (a–i) at an *λ*_ex/em_ of 633/680–740 nm (top) and bright field (bottom). Scale bar, 25 μm. (j) Time course of fluorescence intensity obtained from the Leica software for a–i.

Interesting, the unique caspase-responsive fluorescence of the nanoprobe could be used to evaluate the therapeutic efficiency. To verify this capability, the fluorescent (FITC and Cy5.5) and morphological changes of nanoprobe transfected HeLa cells were tracked in real time under NIR irradiation by confocal fluorescence imaging (see ESI, Fig. S14† and Fig. 8), which showed the increasing luminescence with cell apoptosis. This result proved that the functionalized nanoprobe not only had the potential for *in situ* activation and detection of intracellular caspase, but also could be used for real-time monitoring of the therapeutic effect of a targeted cancer cell, providing a novel tool to evaluate the therapeutic response.

### Monitoring caspase activity in living mouse

**Fig. 9:**
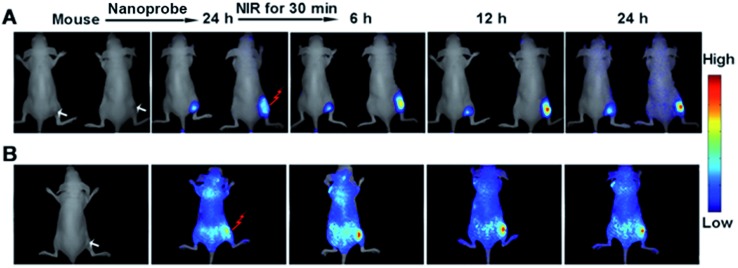
*In vivo* monitoring of the change of casp-3 (A) and casp-9 (B) activities on subcutaneous HeLa tumor-bearing mice after injection with the nanoprobe (100 μL) and irradiation with 808 nm laser for 30 min using the left nanoprobe-injected mouse as control. (A) Excitation: 661 nm; emission: 680–800 nm. (B) Excitation: 455 nm; emission: 500–620 nm.

This design could also be applied to monitor caspase activity in living mouse activated by the treatment effect of the gold nanostructure. A HeLa tumor was subcutaneously implanted on the flank of the nude mice. The tumor-bearing mice were then intravenously injected with the nanoprobe. At 24 h post-injection, tumor accumulation of the nanoprobe was found to be highly efficient (see ESI, Fig. S15†). Afterward, the mice were irradiated with a NIR laser for 30 min to perform the therapy. The therapeutic efficiency was assessed by monitoring the tumor volume after treatment, which showed that the tumor growth was significantly inhibited at 24 h post-irradiation (see ESI, Fig. S16†). The evaluation of excised tissues further demonstrated that strong fluorescence occurred in the cancer tissue in comparison with other organs such as the liver and kidneys (see ESI, Fig. S17†), indicating the cleavage of peptide-9 and/or peptide-3 by the corresponding active caspases. With reduced tumor volume, the fluorescence acquired at both 680–800 nm for Cy5.5 (Fig. 9A) and 500–620 nm for FITC (Fig. 9B) from the irradiated tumor increased gradually after laser irradiation. The more obvious background in Fig. 9B could be attributed to auto-fluorescence at the excitation wavelength of 455 nm, which was greatly reduced under NIR excitation (Fig. 9A). Furthermore, the fluorescence of FITC for casp-9 showed an earlier and greater change (Fig. 9B) than that of Cy5.5 for casp-3 (Fig. 9A), which indicated that casp-9 was activated prior to casp-3, and was in accord with the above cellular experiments. As expected, the un-irradiated mouse showed negligible change in the fluorescence (the left in Fig. 9A) and growing tumor volume. Therefore, the nanoprobe was efficient for the *in situ* activation and monitoring of caspase family activity for therapeutic feedback in living mice.

## Conclusions

This work designs a protocol to monitor *in situ* the evolution of intracellular caspase from upstream to downstream activated by a nanocarrier with a therapeutic effect to inducing cell apoptosis. The evolution is performed by sequentially lighting-up the fluorescence of dyes labelled to peptides assembled on the nanocarrier *via* caspase-catalytic cleavage. The fluorescence signal can be used for not only *in situ* quantification of both caspa-9 and casp-3 activities in cancer cells but also as a self-feedback for the therapeutic response of cancer cells, which leads to a significant method for monitoring therapeutic effect *in vivo*. This methodology is applicable for other nanocarriers with a related effect to monitor caspase-dependent apoptosis. The strategy not only offers a new insight for real-time monitoring the evolution of the intracellular caspase family and evaluating the therapeutic efficacy but also accelerates the uncovering of the biological roles of caspases in cancer apoptosis.
