# Supplementary material for: In situ activation and monitoring of the evolution of the intracellular caspase family
Source: Chem Sci. 2015 Mar 26;6(6):3365–72. doi: 10.1039/c5sc00471c (PMC5490418; doi:10.1039/c5sc00471c)
Supplement: Supplementary file 1 [file SC-006-C5SC00471C-s001.pdf]

Electronic Supplementary Material (ESI) for Chemical Science.
This journal is © The Royal Society of Chemistry 2015

Electronic Supplementary Information

In situ activating and monitoring the evolution of intracellular caspase family

Lei Zhang, Jianping Lei*, Jintong Liu, Fengjiao Ma and Huangxian Ju*

* To whom correspondence should be addressed

Email: jpl@nju.edu.cn, hxju@nju.edu.cn.

Contents

1. Materials and Reagents

2. Apparatus

3. Experimental Section

3.1 Preparation of the nanoprobe

3.2 *In vitro* detection of casp-9 and casp-3 activities

3.3 Cell culture

3.4 Cell extract

3.5 Cytotoxicity evaluation

3.6 Confocal fluorescence imaging and flow cytometric assay

3.7 Immunofluorescent analysis

3.8 Cell apoptosis experiments

3.9 Animal experiments

4. Supporting Figures

5. Supporting References

1. Materials and Reagents

Chloroauric acid ($HAuCl_4$•$4H_2O$), hexadecyltrimethylammonium bromide (CTAB), silver nitrate ($AgNO_3$), sodium borohydride ($NaBH_4$) and ascorbic acid were purchased from Sinopharm Chemical Reagent Co., Ltd. (China). 3-[(3-cholamidopropyl)dimethylammonio]-1-propanesulfonic acid (CHAPS), dicyclohexylcarbodiimide (DCC), N-hydroxysuccinimide (NHS), folic acid (FA), trypsin, bovine serum albumin (BSA), lysozyme and pepsin were purchased from Sigma-Aldrich (USA). HS-poly(ethylene glycol) (PEG)-NH_2 (M.W. 3400) was obtained from JenKem Technology Co. Ltd. (China). Human caspase-9 (casp-9) and caspase-3 (casp-3) recombinant proteins, casp-9 and casp-3 activity assay kits (colorimetric) and cell lysis buffer were purchased from millipore (Billerica, MA, USA). PE Annexin V apoptosis detection kit was purchased from BD Biosciences (USA). Cleaved casp-3 rabbit mAb was purchased from Cell Signaling (USA). Cleaved casp-9 antibody was obtained from ThermoFisher Scientific (USA). 4',6-Diamidino-2-phenylindole (DAPI), LysoTracker Red, FITC conjugated or Cy3 conjugated goat anti-rabbit IgG (H+L) antibody were all purchased from Life Technologies (USA). Casp-3 inhibitor (Z-DEVD-FMK), casp-9 inhibitor (Z-LEHD-FMK) and staurosporine (STS) was purchased from Santa Cruz (USA). 3-(4,5-Dimethylthiazol-2-yl)-2-diphenyltetrazolium bromide (MTT) was obtained from KeyGen Biotech. Co. Ltd. (Nanjing, China). Caspase assay buffer contained 40 mM HEPES (pH 7.4), 100 mM NaCl, 1 mM EDTA, 10% sucrose, and 0.1% CHAPS. Phosphate buffer saline (PBS, pH 7.4) contained 136.7 mM NaCl, 2.7 mM KCl, 8.72 mM Na_2HPO_4 and 1.41 mM KH_2PO_4. All other reagents were of analytical grade. All aqueous solutions were prepared using ultrapure water ($\geq$ 18 MΩ, Milli-Q, Millipore).

Casp-9 specific peptide CLEHDK (FITC-labelled, peptide-9) and casp-3 specific peptide CDEVDK (Cy5.5-labelled, peptide-3) were synthesized and purified by Sangon Biological Engineering Technology & Co. Ltd (Shanghai, China).

2. Apparatus

The transmission electron microscopic (TEM) images were obtained on a JEM-2100 transmission electron microscope (JEOL Ltd., Japan). Zeta potential analysis was performed on a Zetasizer (Nano-Z, Malvern, UK). The UV-vis absorption spectra were obtained with a UV-3600 UV-Vis-NIR spectrophotometer (Shimadzu Co., Kyoto, Japan). Infrared (IR) spectra were recorded on a Nicolet NEXUS870 Fourier transform infrared

(FT-IR) spectrometer (Madison, WI). MALDI-TOF-MS experiments were performed using an Applied Biosystems 4800 proteomics analyzer equipped with a Nd:YAG laser operating at 355 nm, a repetition rate of 200 Hz, and an acceleration voltage of 20 kV. ^{1}HNMR spectra were recorded with a Bruker 500 MHz spectrometer. Flow cytometric analysis was performed on a Coulter FC-500 flow cytometer (Beckman-Coulter). The fluorescence spectra were obtained on a RF-5301PC spectrofluorophotometer (Shimadzu, Japan). MTT and casp-9 & casp-3 activity assays were carried out on a Synergy hybrid 1 multimode microplate reader (BioTek). The cell images were gained on a TCS SP5 laser scanning confocal microscope (Leica, Germany). *In vivo* fluorescence imaging was performed using a Maestro EX in-vivo Imaging System.

3. Experimental Section

3.1 Preparation of the nanoprobe

Gold nanorods (AuNRs) were synthesized as the model nanocarrier by the seed-mediated growth method. Briefly, gold seeds were prepared by adding freshly prepared ice-cold $NaBH_4$ (10 mM, 0.6 mL) into a solution containing CTAB (100 mM, 7.5 mL) and $HAuCl_4$ (10 mM, 0.25 mL), and then stirring for 5 minutes. The resulting gold seed solution was left for 3 h. Meanwhile, growth solution was prepared by mixing CTAB (100 mM, 40 mL), $HAuCl_4$ (10 mM, 1.7 mL), $AgNO_3$ (10 mM, 0.25 mL) and ascorbic acid (100 mM, 0.27 mL). After the seed solution (0.2 mL) was gently added into the growth solution and left for 10 hours, the AuNRs were obtained.

After the prepared AuNR solution was centrifuged twice to remove excess CTAB and concentrated to approximately 2 nM (optical density (OD) = 0.5 at 787 nm), 100 μL of 2 mM HS-PEG-NH_2, 500 μL of 0.1 mM peptide-9 and/or 500 μL of 0.1 mM peptide-3 were added in the solution (100 μL) and incubated for 24 h at room temperature. The reaction solution was then centrifuged at 8000 rpm for 5 min at 25 °C, and the obtained precipitate was washed twice to remove the unbound peptides and HS-PEG-NH_2 as the supernatant. HS-PEG-NH_2, peptide-9 and peptide-3 could be immobilized onto the nanorod surface via the Au-S bond. The resulting peptide-9-AuNRs, peptide-3-AuNRs and peptides-AuNRs were resuspended in ultrapure water for further experiments.

NHS-FA was then prepared with a standard carbodiimide chemistry to couple HS-PEG-NH_2 on the nanorods.[1-3] 100 mg FA dissolved in 10 ml of dry dimethyl sulfoxide (DMSO) was reacted with DCC (50 mg) and NHS (53 mg) in the presence of 0.06 mL of

triethylamine overnight at room temperature. After the by-product was removed by filtration, the DMSO solution was concentrated under reduced pressure, and NHS-FA was precipitated in ether. The product was then washed several times with anhydrous ether, dried under vacuum, and stored as a yellow powder. NHS-FA was characterized through IR, MS and ^{1}HNMR analysis, which showed a mass of 535.18 (calculated at 535.47 for NHS-FA – 3H$^+$) and the δ (ppm) values in ^{1}HNMR (500 MHz, DMSO): 8.67–8.65 (m, 2H), 7.64 (d, J=10.0 Hz, 2H), 6.91 (t, J=5.0 Hz, 1H), 6.65 (d, J=10.0 Hz, 2H), 4.52–4.48 (m, 3H), 2.99–2.95 (m, 5H), 2.81 (s, 1H), 2.60 (s, 1H), 2.55 (s, 2H), 2.31 (t, J=5.0 Hz, 2H).

After 10 μL of NHS-FA (0.1 M, in DMSO) was added to 500 μL of the prepared peptide-9-AuNRs, peptide-3-AuNRs or peptides-AuNRs ($OD_{787\ nm}$ = 0.5), the mixture was adjusted to pH 11 and reacted at room temperature for 15 min. The reaction solution was then centrifuged at 8000 rpm for 5 min at 25 °C to get the precipitates. After washed twice to remove the unbound NHS-FA, the obtained precipitates were resuspended in PBS as the nanoprobe, nanoprobe-9 and nanoprobe-3, individually. The OD values of these nanoprobes at 787 nm were all 0.5 unless noted otherwise.

3.2 *In vitro* detection of casp-9 and casp-3 activities

10 μL nanoprobe ($OD_{787\ nm}$ = 0.5) was incubated with recombinant caspase proteins in caspase assay buffer (100 μL) at 37 °C for the designed times, and the change of fluorescence intensity was measured. The fluorescence spectra were collected from 500 to 650 nm under an excitation at 490 nm and from 680 to 900 nm under an excitation at 675 nm for casp-9 and casp-3, respectively.

3.3 Cell culture

HeLa, HaCat and MCF-7 cell lines were obtained from KeyGen Biotech (Nanjing, China). HeLa, HaCat and A549 cells were cultured in a flask in Dulbecco's modified Eagle's medium (DMEM, Gibco) and MCF-7 cell lines were cultured in Roswell Park Memorial Institute (RPMI)-1640 (Gibco), supplemented with 10% fetal bovine serum (FBS, Gibco), penicillin (100 μg mL^{-1}), and streptomycin (100 μg mL^{-1}) at 37 °C in a humidified atmosphere containing 5% CO_2. Cell numbers were determined with a Petroff-Hausser cell counter (USA).

3.4 Cell extract

After HeLa cells were treated with STS (2 μM, a widely used apoptosis inducer) for 2 h,

2.0×10^6 cells were dispensed in a 1.5-mL Ependorff tube, washed twice with ice-cold PBS (0.1 M, pH 7.4), and resuspended in 200 μL of ice-cold caspase lysis buffer containing pH 7.4 HEPES-NaOH, 0.1% sucrose, 1% CHAPS, 2 mM EDTA. The mixture was incubated for 30 min on ice and centrifuged at 10000 rpm for 5 min at 4 ^{o}C to obtain the supernatant as cell extract for caspase analysis.

3.5 Cytotoxicity evaluation

The cytotoxicity of the nanoprobe and NIR irradiation was individually studied by MTT assay. Briefly, HeLa cells (100 μL, 1.0×10^5) were seeded in the wells of 96-well plate for 12 h, washed twice with PBS, and then incubated with 200 μL culture medium containing different amounts of the nanoprobe ($OD_{787\ nm} = 0.5$) or/and exposed to NIR irradiation. Meanwhile, the cells were incubated with 200 μL culture medium without the nanoprobe as control. After washing with PBS, MTT (50 μL, 1 mg mL^{-1}) was added to each well, and incubated for 4 h at 37 °C. The medium was then removed, and 150 μL DMSO was added to each well. After the cell plate was vibrated for 15 min at room temperature to dissolve the crystals formed by the living cells, the absorbance of each well was measured at 490 nm. The relative cell viability (%) was calculated by $(A_{\text{test}}/A_{\text{control}}) \times 100$.

3.6 Confocal fluorescence imaging and flow cytometric assay

HeLa cells were seeded in 35-mm confocal dishes (Glass Bottom Dish) at a density of 5.0 $\times 10^4$ per dish and incubated for 12 h at 37 °C. The medium was then replaced with fresh culture medium containing 10 μL the nanoprobe to incubate for 3 h. After NIR irradiation for different times and washing with PBS, the fluorescence of cells was collected from 500 to 560 nm and from 680 nm to 740 nm on a confocal laser scanning microscope with the excitation wavelength of 488 nm and 643 nm for FITC and Cy5.5, respectively. All images were digitized and analyzed with Leica Application Suite Advanced Fluorescence (LAS-AF) software package. The flow cytometric assay was performed over both FL1 (FITC) and FL4 (Cy5.5) channels after HeLa cells (5.0×10^5) were incubated with the nanoprobe (50 μL) for 3 h and then treated with NIR for different times.

3.7 Immunofluorescent analysis

After incubated with the nanoprobe-3 or the nanoprobe-9 (10 μL) for 3 h and treated with NIR irradiation for different times, HeLa cells were fixed in confocal dishes by 4% formaldehyde, washed with PBS and treated with 2% BSA in PBS to quench free

aldehyde and block nonspecific binding. After washed twice with PBS, the cells were further incubated with anti-caspase primary antibody (1:100) for 1 h at room temperature, washed with PBS and incubated with dye-conjugated goat anti-rabbit IgG (1:400) for 30 min. After stained with DAPI dye, the cells were imaged. For the specific imaging of intracellular caspase-9/-3, nanoprobe-9/-3 was used instead of the nanoprobe.

3.8 Cell apoptosis experiments

Briefly, 5.0×10^5 HeLa cells were seeded in a 6-well plate for 12 h containing 2 mL fresh DMEM in each well. These cells were then incubated with nanoprobe (50 μL) for 3 h and then with NIR irradiation for different times. The resulting cells were collected, stained with the mixture of 5.0 μL Annexin V-PE and 5.0 μL 7-amino-actinomycin D (7-AAD) for 15 min, and analyzed with flow cytometry over FL2 (Annexin V-PE) and FL3 (7-AAD) channels.

3.9 Animal experiments

Specific pathogen-free female BALB/c nude mice, 5−6 weeks of age, were purchased from Shanghai Laboratory Animal Center, Chinese Academy of Sciences and bred in an axenic environment. All animal operations were in according with institutional animal use and care regulations approved by the Model Animal Research Center of Nanjing University (MARC). HeLa tumor model was established by subcutaneous injection of HeLa cells (1.0×10^6) into the selected position of the nude mice. During treatment, mice were anesthetized with 2.5% isoflurane in oxygen delivered at a flow rate of 1.5 L min^{-1}. For time-dependent *in vivo* imaging, one HeLa tumor-bearing mouse was intravenously injected with 100 μL nanoprobe (~10% injection dose g^{-1}) via tail vein and conducted on a Maestro EX in-vivo Imaging System at 6, 16, 24 and 48-h postinjection (p.i.). For monitoring of caspase activity, two HeLa-tumor bearing mice were intravenously injected with 100 μL nanoprobe via tail vein. After 24-h p.i, one of them was treated with NIR irradiation for 30 min, and they were then used to perform *in vivo* fluorescence imaging. During the image acquisition process, the mice were also anesthetized with 2.5% isoflurane in oxygen delivered at a flow rate of 1.5 L min^{-1}. *In vivo* therapeutic efficiency was studied by measuring the tumor volume using a vernier caliper after treatment. The greatest longitudinal diameter (length) and the greatest transverse diameter (width) of each tumor were determined using a vernier caliper, and the tumor volume was calculated using length × width^2 × 0.5.[4] After the *in vivo* imaging, the mice were euthanized to obtain the organs and tumor tissue for imaging.

4. Supporting Figures

Characterizations of the nanoprobe

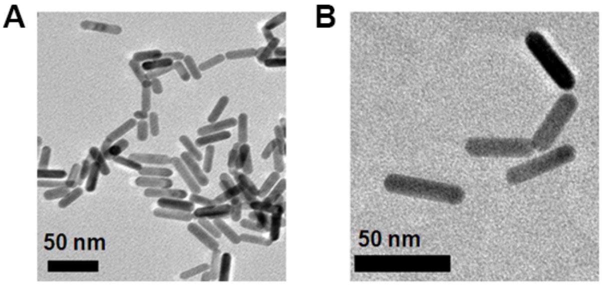

Fig. S1. TEM images of (A) gold nanorods and (B) the functionalized nanoprobe.

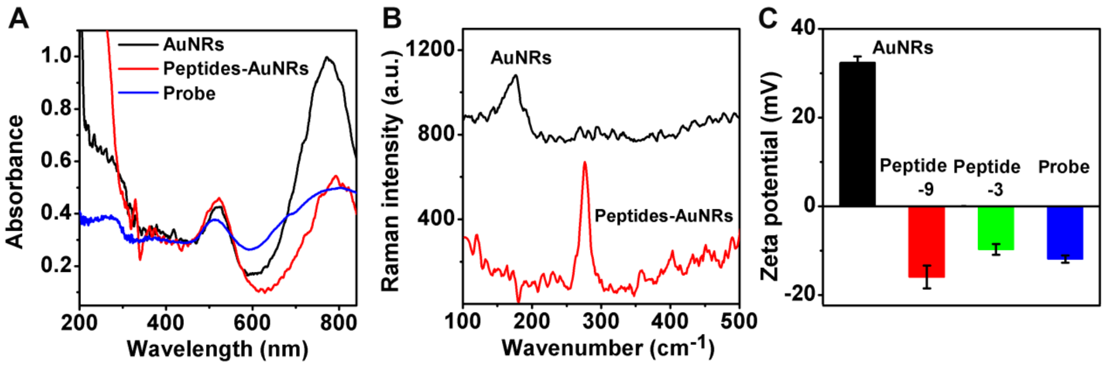

Fig. S2. (A) UV-vis spectra of raw nanorods (AuNRs), peptides functionalized nanorods (peptides-AuNRs) and the nanoprobe. (B) Raman spectra of raw AuNRs and peptides-AuNRs. (C) Zeta potentials of raw AuNRs, peptide-9, peptide-3 and the nanoprobe.

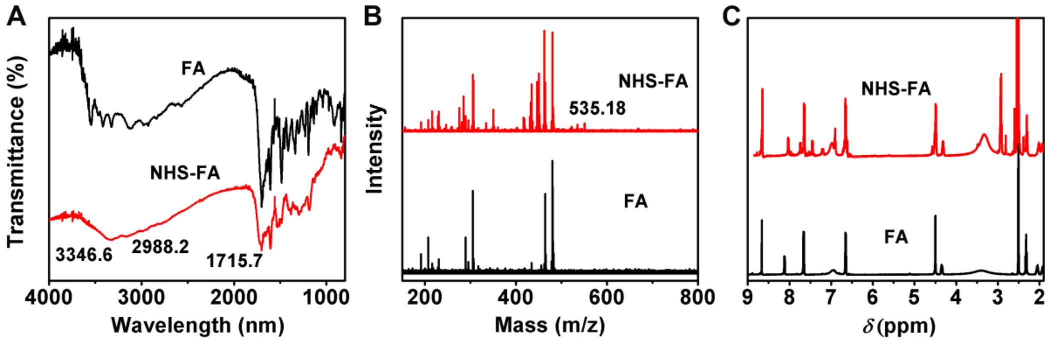

Fig. S3. FT-IR (A), mass (B) and ^{1}HNMR (C) spectra of FA and NHS-FA.

Quenching effect of the nanorod on FITC and Cy5.5

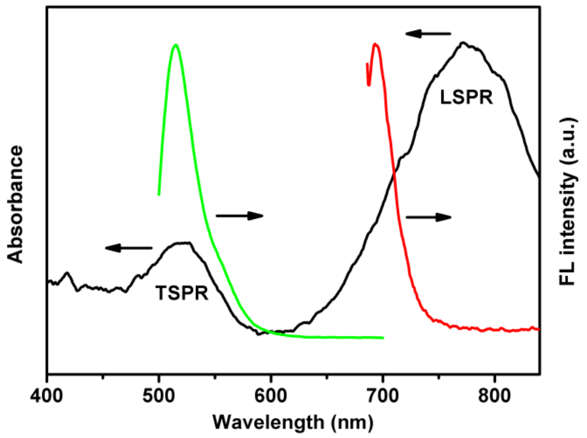

Fig. S4. Absorption spectrum (black) of gold nanorod and fluorescence spectra of FITC (green) and Cy5.5 (red). TSPR and LSPR represent transverse and longitudinal SPR absorption of gold nanorod, respectively.

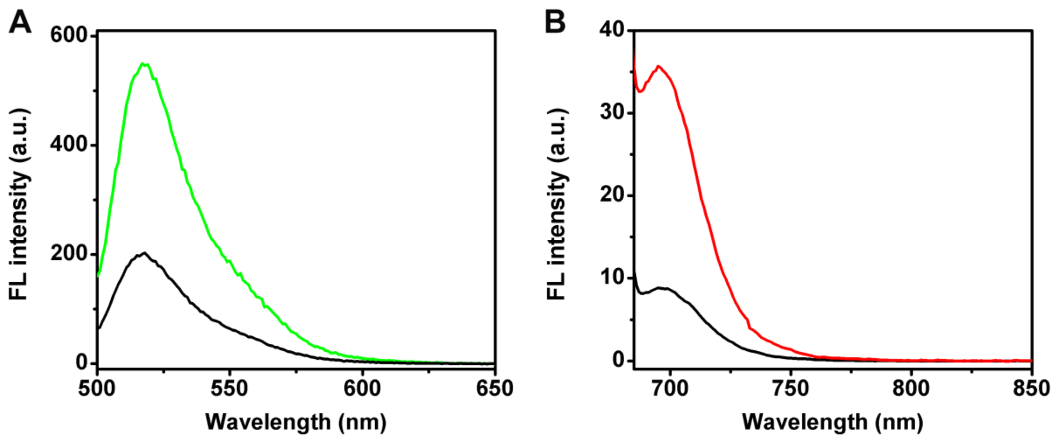

Fig. S5. Fluorescence spectra of (A) peptide-9 (green) and peptide-9 functionalized gold nanorod (black) with the excitation wavelength of 490 nm, and (B) peptide-3 (red) and peptide-3 functionalized gold nanorod (black) with the excitation wavelength of 675 nm.

Amounts of peptides loaded on the nanocarrier

The loading of peptides on the gold nanocarrier was determined by fluorescence measurement of the labeled dyes in the supernatant obtained from the preparation of the nanoprobe. The standard curves were obtained with known concentrations of peptide-9 and peptide-3.

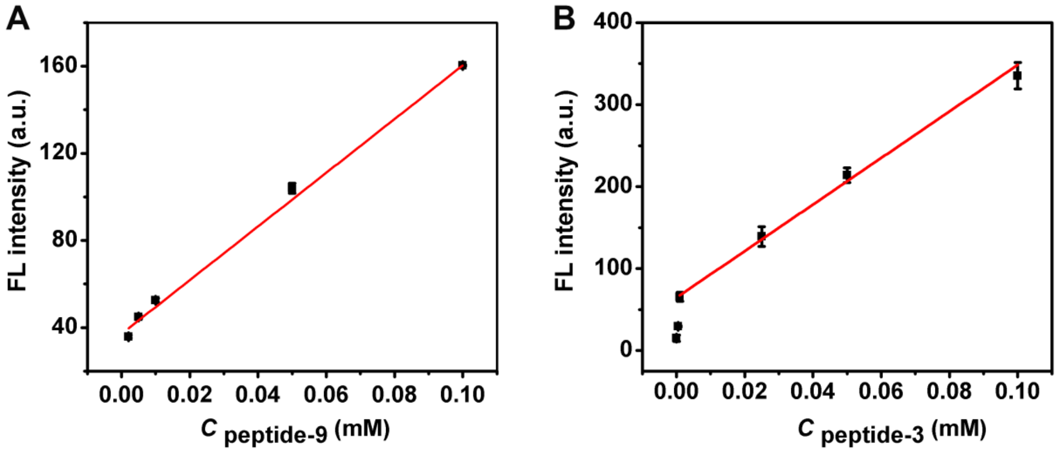

Fig. S6. Plots of fluorescence intensity *vs.* the concentration of (A) peptide-9 and (B) peptide-3.

Cytotoxicity evaluation

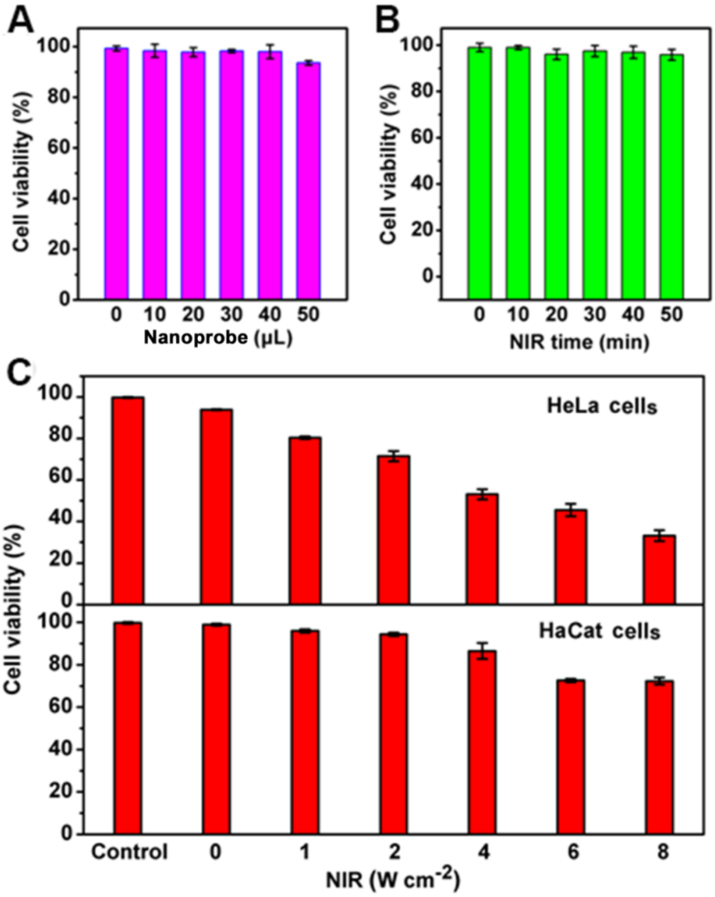

Fig. S7. Viability of HeLa cells after (A) incubation with different amounts of the nanoprobe for 3 h, (B) NIR irradiation for different times and (C) incubation with nanoprobe (10 μL) for 3 h and then with NIR irradiation for 10 min with different power densities.

FA receptor-mediated endocytosis

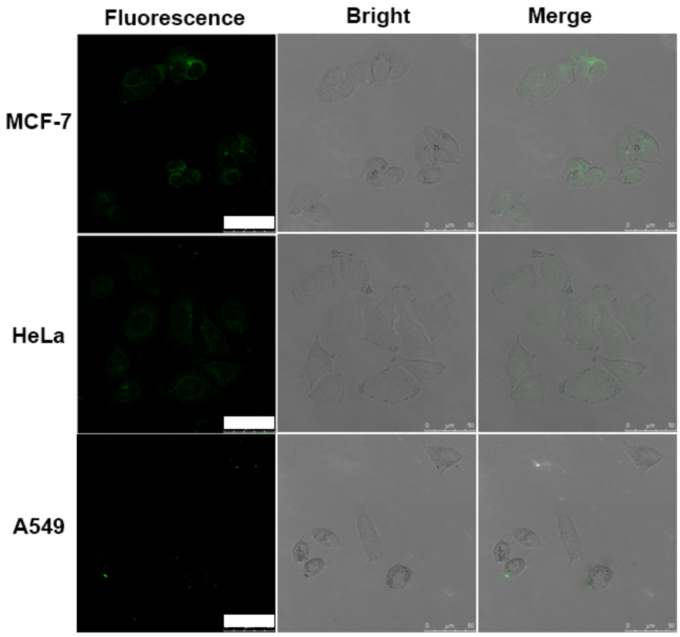

Fig. S8. Confocal fluorescence images of MCF-7, HeLa and A549 cells after transfected with nanoprobe (20 μL) at 37 ^{o}C for 3 h with $\lambda_{ex/em}$ of 488/500–560 nm. Scale bars: 25 μm.

Probe localization in cells

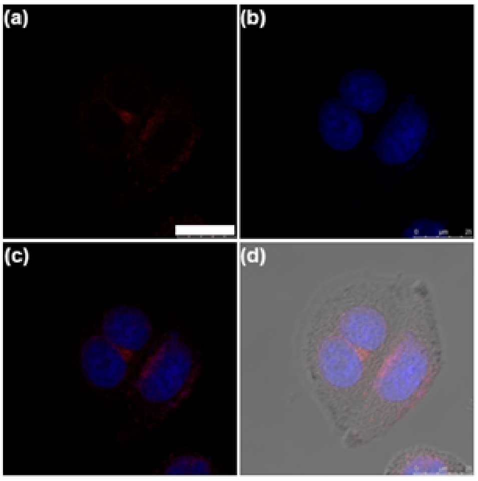

Fig. S9. Confocal fluorescence images of HeLa cells after (a) incubated with nanoprobe (20 μL) at $\lambda_{ex/em}$ of 488/500–560 nm, (b) stained with DAPI (5 μg mL^{-1}) to specifically label cell nucleus at $\lambda_{ex/em}$ of 405/440–480 nm, (c) merge of (a) and (b), and (d) merge of (c) and bright image. Scale bar: 25 μm. Here, the red color represented the FITC fluorescence to distinguish from the blue color of DAPI fluorescence.

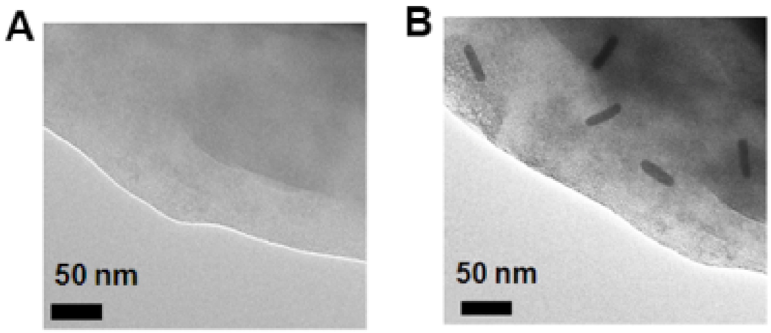

Fig. S10. (A) HeLa cells and (B) HeLa cells transfected with the nanoprobe.

Mitochondrial pathway of apoptosis

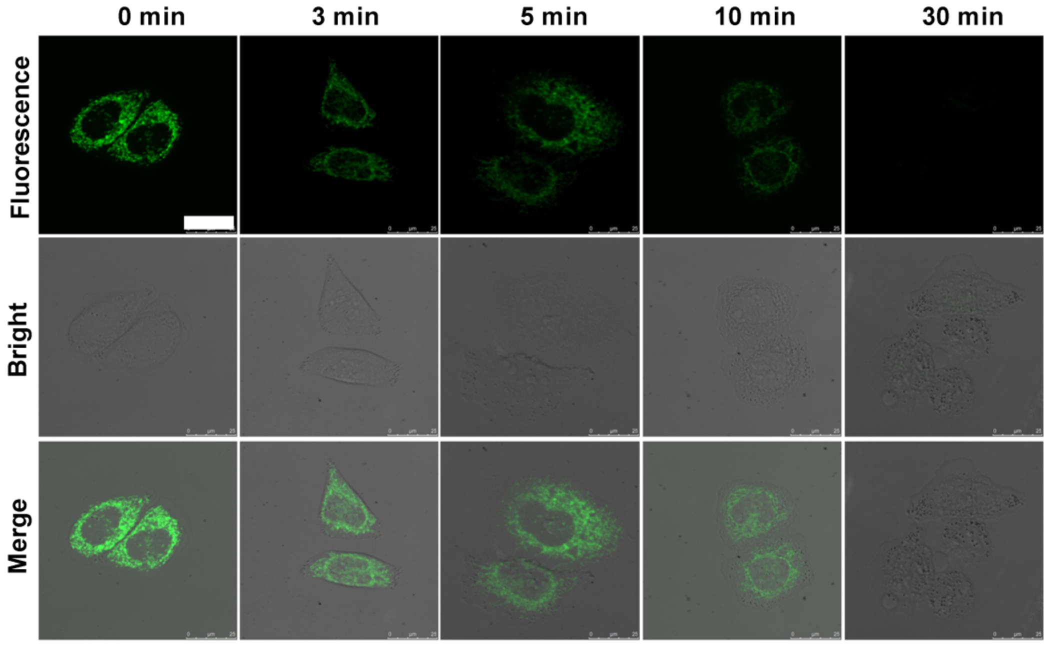

Fig. S11. Confocal fluorescence images of HeLa cells incubated with nanoprobe-3 (10 μL) for 3 h and then stained living mitochondria in cells with Rhodamine 123 (5 μg mL^{-1}) after NIR irradiation for different times at $\lambda_{ex/em}$ of 488/500–560 nm. Scale bar, 25 μm.

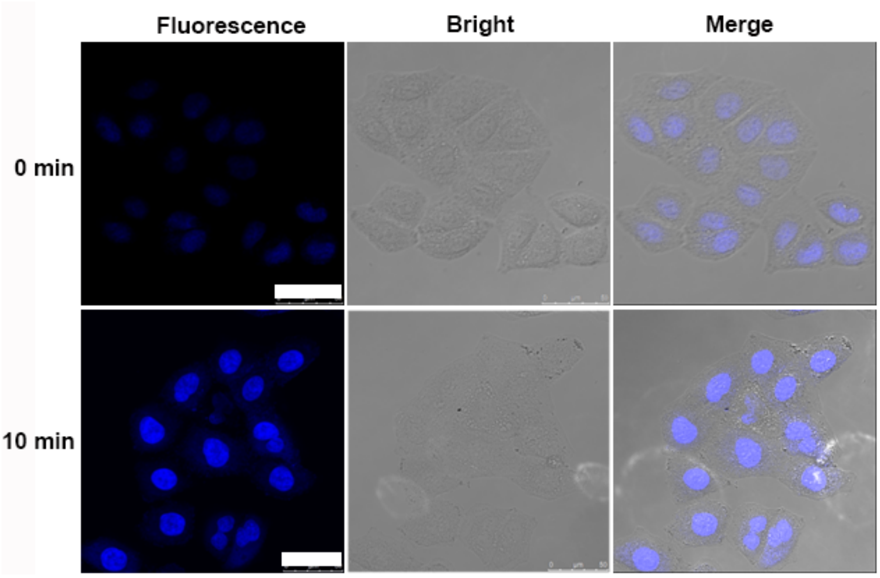

Fig. S12. Confocal fluorescence images of HeLa cells incubated with nanoprobe (10 μL) and then stained with DAPI (5 μg mL^{-1}) before and after NIR irradiation for 10 min at $\lambda_{ex/em}$ of 405/440–480 nm. Scale bars: 50 μm.

Immunofluorescence imaging of HeLa cells

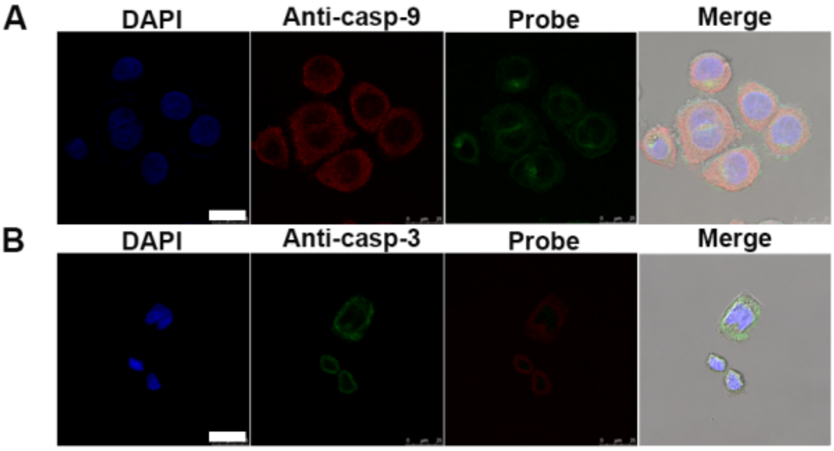

Fig. S13. Confocal fluorescence images of HeLa cells after sequentially treated with (A) nanoprobe-9 (10 μL), NIR irradiation for 10 min, anti-casp-9 antibody, Cy3-labelled secondary antibody and DAPI, and (B) nanoprobe-3 (10 μL), NIR irradiation for 20 min, anti-casp-3 antibody, FITC-labelled secondary antibody and DAPI. $\lambda_{ex/em}$ for DAPI: 405/440–480 nm; $\lambda_{ex/em}$ for anti-casp-9: 541/560-600 nm; $\lambda_{ex/em}$ for anti-casp-3 and nanoprobe-9: 488/500-560 nm; $\lambda_{ex/em}$ for nanoprobe-3: 633/680–740 nm. Scale bars, 25 μm.

Real-time therapy monitoring with the nanoprobe

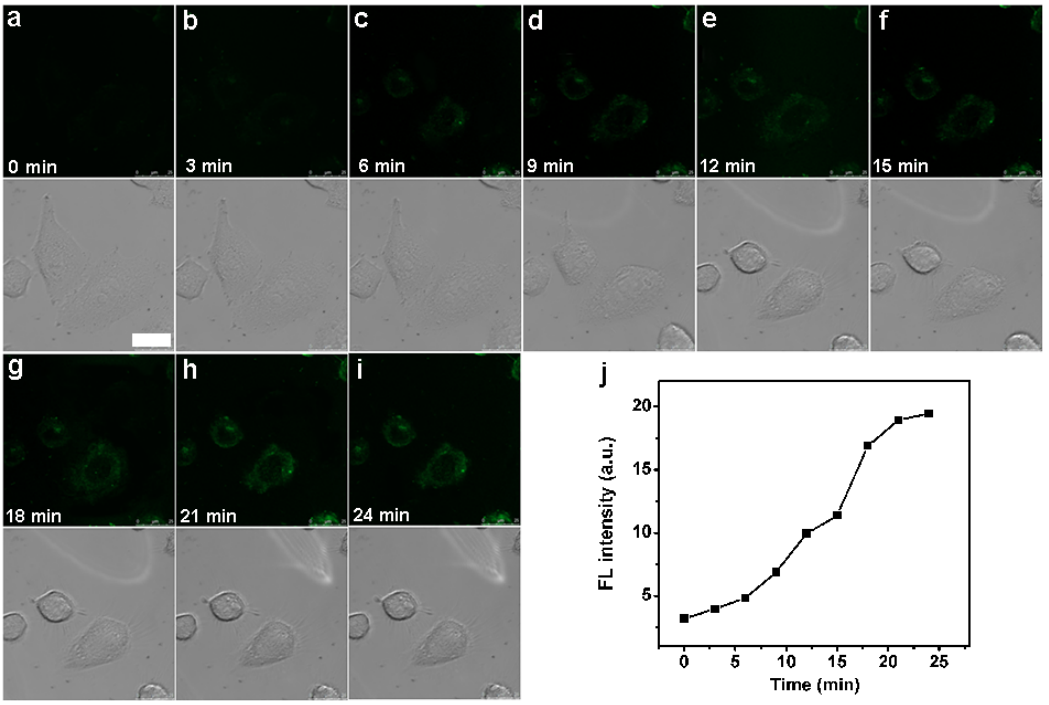

Fig. S14. Real-time monitoring of fluorescence and morphology of HeLa cells treated with nanoprobe (10 μL) for 3 h and then NIR irradiation at a power density of 4 W cm^{-2} for different times (a-i) at $\lambda_{ex/em}$ of 488/500–560 nm (top) and bright field (bottom). Scale bar, 25 μm. (j) Time course of fluorescence intensity obtained from the Leica software for a-i.

Time-dependent *in vivo* fluorescence imaging

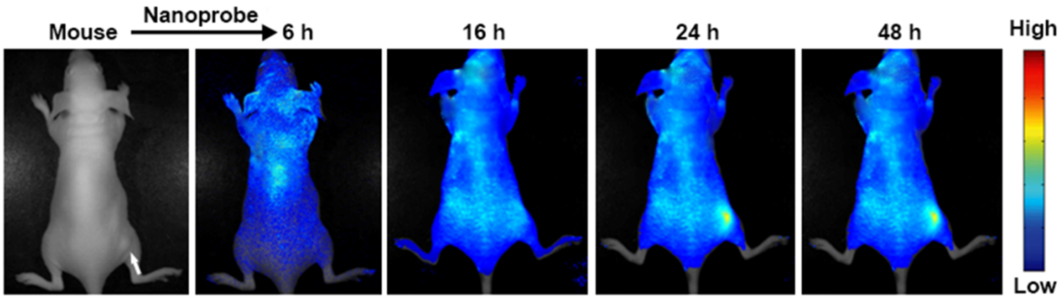

Fig. S15. Time-dependent *in vivo* fluorescence images of subcutaneous HeLa tumor-bearing mouse treated with injection of nanoprobe (100 μL) at $\lambda_{ex/em}$ of 455/500–620 nm.

Change of tumor volume after treatment

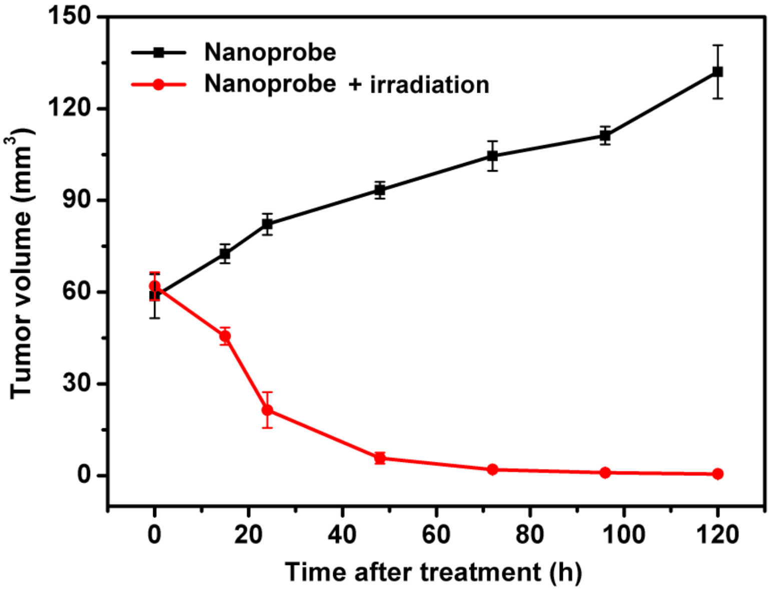

Fig. S16. Change of tumor volume after treatment with 24-h postinjection of nanoprobe (100 μL) and then with or without NIR irradiation for 30 min.

Ex vivo evaluation of excised tissues

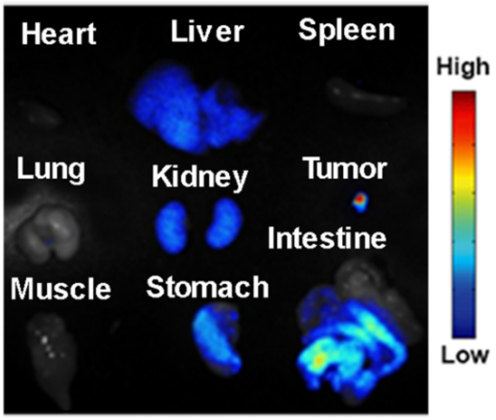

Fig. S17. *Ex vivo* fluorescence image of organs and cancer tissue at 24-h post irradiation from HeLa tumor-bearing mouse treated with 24-h postinjection of nanoprobe (100 μL). Here, the fluorescence in intestine originates from the food for specially feeding the nude mice.

5. Supporting References

1 P. Singh, U. Gupta, A. Asthana and N. K. Jain, *Bioconjugate Chem.*, 2008, **19**, 2239–2252.

2 Y. Q. Zhang, Y. H. Sun, X. P. Xu, X. Z. Zhang, H. Zhu, L. L. Huang, Y. J. Qi and Y. M. Shen, *J. Med. Chem.*, 2010, **53**, 3262–3272.

3 D. M. Euhus, C. Hudd, M. C. LaRegina and F. E. Johnson, *J. Surg. Oncol.*, 1986, **31**, 229–234.

4 R. J. Lee and P. S. Low, *J. Biol. Chem.*, 1994, **269**, 3198–3204.
